# Disinfectants

**Published:** 1893-05

**Authors:** Carrie M. Stewart


### Disinfectants.

BY CARRIE M. STEWART, D.D.S.

By the term disinfectants, is commonly understood any agent which will destroy the life of germs. It may, however, possess a broader meaning, and include deodorants, detergents and germicides; each of these being limited in its action or partaking of the nature of the other two. For instance, a typical germicide may not be a good deodorant or detergent, while a good detergent, as $H^2 O^2$, may not be a germicide in the true sense of the word, but more of the nature of an antiseptic in its action upon bacteria.

The experiments given in this paper, were made for the purpose of ascertaining what agent, alone or combined, would best answer the requirements for a dental disinfectant; one which can be used in diseased conditions of the oral cavity, alveolar abscess, putrid pulp, etc., with especial reference to quickness of action, with the least possible irritation and greatest acceptibility to the patient.

The germs used in experimentations were those often found in the mouth, the staphylcoccus pyogenes aurens and allus. The bacteria belonging to the cocci groupi were selected because of the greater resistance of this form of germ to destructive influences than those belonging to the bacilli or spirilli types.

The germs were grown in test tubes containing 5 c.c. of beef bouillon. To this 1 c.c. of the disinfectant was added. The exact amount of each being determined in order to be sure of the amount of dilution of the reagent used, and also because of the greater perfection of the drug's destructive action, it being subjected to a more severe test when tried upon the millions of micro-organisms growing in the bouillon, than if the bacteria had been introduced into the germicidal solution with a platinum wire, on dried threads or a few drops of the inoculated medium. Besides, in the number of germs and dilution of the agent, it more nearly approximates the condition found in the mouth.

The time used was fifteen minutes, gelatine plates being inoculated at the end of 1, 2, 3, 5, 10, 15 minutes respectively. This time is the same, as near as possible, as that used ordinarily in the employment of medicinal agents in the mouth.

The loop of platinum wire used for inoculating the gelatine was very small, in order to avoid carrying over enough of the disinfectant to form an area of sterilized gelatine around the germs, and preventing their growth, give rise to the false impression that they were deprived of life before being carried over. The liquified gelatine was thoroughly shaken, after the introduction of the disinfected germs, for the same reason.

The normal time for the development of bacteria which had not been acted upon by chemical agents, was usually about forty-eight hours with the temperature of the room between 25° and 30° centigrade.

Heat, of course, is the most effective form of sterilization, and although it cannot be conveniently applied in the mouth, its effects upon the germs were proven and found that in boiling water the staphylcoccus pyogenes aurens were destroyed in one minute. With steam heat at 100° the same results followed in the same length of time.

From seven to eight days was the time given for the development of the plates, as only the most resistant organisms survive the exposure to destructive agents, and the longer the time employed the greater number succumb, there being a "survival of the fittest" here as elsewhere in Nature's economy. Those which finally develop require more time for the process than would be necessary under the natural conditions. Bichloride of mercury, 1 to 2,000, with the pyogenes aurens, bouillon culture seven days old. Development was prevented by one minute's exposure.

There has, however, been some dispute in regard to the high rank given Hg $Cl_2$ as a germicide, some claiming that although it retards the development of germs for an indefinite period of time, under favorable circumstances and the action of certain salts, the chemical combination formed between the albuminous capsule of the germ and the bichloride of mercury will be dissolved and development take place.

It is well known that an extremely small amount of this salt is at least antiseptic in character, and although this theory has not been satisfactorily proven as yet, neither has it been disproven, and on this account further experimentation with the salt was left until another time.

Oil of peppermint 1 c.c. to 5 c.c. of a bouillon culture of the aurens. Development of an innumerable number of colonies took place in every plate after a period of three days.

Permanganate of potassium, 1 per cent solution, 1 to 5 of bouillon culture of aurens. Numerous development occurred after three days. In a two per cent solution in the same proportions the same results were obtained in the same time. After a fifteen minutes exposure, the development in both the one per cent and two per cent solutions was somewhat retarded.

Permanganate of potassium four per cent, 1 to 5 of a bouillon culture of pyogenes allus. Development took place in all the plates after three days.

Permanganate of potassium, 1 to 5 of the concentrated solution, with the aurens. No development occurred in any plate.

Chloro-phenique, bouillon culture of albus, 1 to 5. No development occurred This agent precipitates albumen and is irritating to the tissues.

Listerine, 1 to 5 of a bouillon culture of the albus. All plates were well developed after four days.

Saccharine prevented the development of the pyogenes aurens, in the usual proportions, after a one minute exposure.

The action of carbolic acid being so well known, no experimentations were made with this drug.

Some of the other products of coal tar distillation were next tried, the concentrated liquids coming from the German laboratories and the desired percentage made with distilled water. A two per cent solution was employed.

Aseptol, a clear, slightly brown liquid, with a decidedly acid reaction, 1 cc. to 5 cc. of a bouillon culture of the aurens. After five days, great development took place in every plate but that of the two minutes' exposure, which presented no colonies. This irregularity may be accounted for by the fact that only feebly resistant germs happened to be transferred to the second plate, and destruction having been accomplished before the transference, there was no development; or it may be, too much of the aseptol was carried over, although that is hardly likely as every means was employed to prevent this taking place.

Toluol, being extremely greasy and possessing a pronounced kerosene odor, was not tried.

Creolin, a very dark solution with a strong odor of tar, produced a pinkish white emulsion with water and alcohol. 1 to 5 cc. of a bouillon culture of the albus, prevented all development.

Creosol, a beautiful straw colored liquid, neutral reaction and sharp biting taste, in the usual proportions, with the albus, prevented all development.

Chinolin, a golden brown liquid, producing an emulsion with water, 1 to 5 cc. of a bouillon culture of the albus. Development took place in the first three plates after seven days. The one minute plate being developed in five days. The agent appeared to be a germicide after a five minutes application.

Lysol, a dark brown syrupy liquid, in the concentrated state, produces a beautiful straw colored solution in distilled water or alcohol, while water containing lime salts assumes a decidedly milky appearance by the addition of this agent. It possesses a characteristic odor which is not at all unpleasant, but rather agreeable. A one per cent solution was found to be an antiseptic only, development appearing after three days on the first two plates and a day or two later on the others. From a two per cent solution upwards the agent is germicidal in its action in one minute. On the germs of saliva, no development took place in the plate treated with lysol, while eight colonies resulted in the plate of normal saliva.

Lysol more nearly answers the definition of a disinfectant than any agent experimented with, as it is a prompt and effectual deodorizer, a good detergent and a prompt germicide; combining in one drug the qualities so essential for a dental disinfectant.

When used on very sensitive surfaces, the weaker solutions should be used first, as irritation results from the immediate application of a five per cent or six per cent solution to such surfaces.

In addition to its prompt, beneficial action, lysol is not disagreeable to most patients, and seemingly uninjurious to the teeth.

There are disinfectants and disinfectants and this paper presents a glimpse of a few out of hundreds. These few, however, have been thoroughly and carefully tried and the results given. The point now-a-days is to find something one is sure of and can rely upon.

New discoveries are constantly being made and old remedies discarded, until it becomes quite a problem to the practitioner what to depend upon. Only yesterday, comparatively, iodoform was relied upon as one of the great mainstays of dentistry, but it has been forced to take a lower position in the scale of dental medicine, and its place will probably be filled, in the near future, by something far better.

It is useless to have a great number of remedies, the action of none of which we thoroughly understand, perhaps, but all being highly recommended they are supposed to be good in almost any emergency, and when the emergency arises the practitioner is at a loss just which one of his valuable medicaments to employ. It would be far better and save a great loss of time, energy and general dissatisfaction, if each one would thoroughly acquaint himself with a limited number of agents, satisfy himself in regard to their efficacy in certain conditions, and he will then have at hand something he can rely upon in time of need when everything else seems to be doubtful and uncertain. Of course this does not altogether prevent the trial of new remedies, but gives one a tried list to fall back upon when an urgent case is presented, which is much more satisfactory than uncertain experimentation all of the time.

